# Differentiating Contact with Symptomatic and Asymptomatic Infectious Individuals in a SEIR Epidemic Model

**DOI:** 10.1007/s11538-025-01416-2

**Published:** 2025-02-04

**Authors:** Victoria Chebotaeva, Anish Srinivasan, Paula A. Vasquez

**Affiliations:** Department of Mathematics, University of South Carolina, 1523 Greene St, Columbia, SC 29208 USA

**Keywords:** SEIR model, Erlang distribution, Asymptomatic transmission, Infectious disease modeling

## Abstract

This manuscript introduces a new Erlang-distributed SEIR model. The model incorporates asymptomatic spread through a subdivided exposed class, distinguishing between asymptomatic ($\hbox {E}_a$) and symptomatic ($\hbox {E}_s$) cases. The model identifies two key parameters: relative infectiousness, $\beta _{{SA}}$, and the percentage of people who become asymptomatic after being infected by a symptomatic individual, $\kappa$. Lower values of these parameters reduce the peak magnitude and duration of the infectious period, highlighting the importance of isolation measures. Additionally, the model underscores the need for strategies addressing both symptomatic and asymptomatic transmissions.

## Introduction

Despite significant advancements in medicine, humanity continues to encounter many challenges in the battle against different diseases. Recent events have underscored the continued vulnerability of humans to the rapid propagation of infectious diseases and the ensuing epidemics. The 2002–2004 severe acute respiratory syndrome (SARS) outbreak (Knobler et al. 2004), the 2009 swine flu pandemic (https://www.cdc.gov/flu/pandemic-resources/2009-h1n1-pandemic.html), the West African Ebola epidemic, the Kivu Ebola epidemic (https://www.cdc.gov/vhf/ebola/outbreaks/drc/overview.html), and the global COVID-19 (Holmes et al. 2020; Nicola et al. 2020; Chinazzi et al. 2020; Kraemer et al. 2020) pandemic have demonstrated the ability of viruses to spread over large areas and affect people, healthcare systems, disrupting economies and the daily lives of people around the world. At the same time, mathematical models have emerged as valuable instruments in comprehending the intricate dynamics of disease transmission. Models aid in forecasting the spread of diseases, diminishing uncertainty, and expediting decision-making procedures. While no singular model can entirely grasp the complexities of disease propagation, their usefulness is widely acknowledged. Therefore, continuously improving epidemiological models is crucial for enhancing disease control strategies.

In 1927, McKendrick and Kermack published the first modern mathematical study using such models (Kermack and McKendrick 1927). Their work, focused on infectious and susceptible (SI) compartments, laid the foundation for understanding disease dynamics in populations over time. Subsequent developments, including the susceptible-infected-recovered (SIR) model and its variants, have made significant contributions to epidemiology. These models and modifications incorporating disease control measures continue to play a critical role in studying the dynamics of infectious diseases (Hethcote 2000).

Introducing additional classes expanded the SIR model to include the exposure class (E), resulting in the SEIR model. This model allows for a more accurate representation of the spread and dynamics of infectious diseases. By incorporating the exposure class, the SEIR model captures the incubation period and the potential for pre-symptomatic transmission, which are crucial factors in understanding and controlling disease outbreaks.

Subsequent modifications include the Erlang-distributed SEIR model. This model improves upon the standard SEIR model by providing a more realistic representation of the latent and infectious periods of a disease. Standard SEIR models assume that transitions between compartments (exposed to infectious, and infectious to recovered) follow exponential distributions. This implies a limited degree of variability in the duration of these periods, failing to capture the wide range of durations often observed in real-world infections, where some individuals may remain in a given stage for significantly longer or shorter periods than average. In contrast, the Erlang distribution, by allowing for a more flexible distribution shape, can better represent the observed heterogeneity in these durations, enabling the model to capture the higher variability seen in latent and infectious stages.

In this manuscript, our aim is to explore various modifications and refinements to the Erlang-distributed SEIR model which enhance its effectiveness in predicting and managing infectious diseases. In particular, we explore the effects of distinguishing between asymptomatic and symptomatic transmission in infectious diseases. Asymptomatic individuals are defined as those who do not show any symptoms of an infection. However, as suggested by Peirlinck et al. (2020), individuals who show mild symptoms that are uncharacteristic of the disease may also be classified as asymptomatic.

Recent data has shown that asymptomatic and pre-symptomatic infected individuals played a key role in the transmission dynamics of COVID-19 pandemic (Gatto et al. 2020; Das et al. 2021; Pei et al. 2022; Khairulbahri 2023). Consequently, numerous studies have focused on modeling these aspects of disease spread. However, challenges in data collection for asymptomatic cases limit our understanding of their precise impact. Furthermore, factors such as vaccination can influence the ratio of symptomatic to asymptomatic individuals, complicating the epidemiological picture. It is also more difficult to contain a disease with a higher percentage of asymptomatic individuals. Nevertheless, the significance of asymptomatic cases in disease transmission cannot be overstated.

To effectively address these complexities, epidemiological models must accurately capture the dynamics of asymptomatic transmission. For instance, Grunnill (2018) emphasize the importance of incorporating targeted vaccination strategies into SEIR models to control outbreaks. The challenge is magnified for diseases with a high proportion of asymptomatic cases, which can rapidly disseminate as diseases with more asymptomatic individuals tend to have a higher reproductive number, $R_0$Peirlinck et al. (2020).

To better understand the dynamics of such diseases, Anggriani et al. (2022) developed a model differentiating symptomatic and asymptomatic dengue fever cases. The authors analyzed outbreak potential and proposed a critical immunity level – the minimum population needing protection, via vaccination or prior infection, to prevent outbreaks. The model showed this critical level depends on how easily the virus jumps between symptomatic and asymptomatic cases and how long immunity lasts. Leung et al., Leung et al. (2018) further refined this understanding by introducing a time-varying ratio of symptomatic to asymptomatic cases, emphasizing the need for adaptable modeling approaches.

Castañeda et al. (2023) expanded on this by creating a model explicitly separating asymptomatic and symptomatic infectious individuals. Their work demonstrates the advantages of this type of modeling in informing disease control strategies.

Furthermore, the substantial impact of asymptomatic transmission is evident in the study by Chowdhury et al. (2022). Their findings show that during the first year of the COVID-19 pandemic, asymptomatic transmission had a more significant impact on disease spread than symptomatic transmission. This underscores the necessity of comprehensive models that account for asymptomatic spread of the disease.

This manuscript introduces a novel Erlang-distributed SEIR model to investigate the impact of asymptomatic transmission, seasonality, and waning immunity on infectious disease dynamics. By incorporating separate compartments for asymptomatic and symptomatic individuals during the latent and infectious periods, the model provides a more nuanced understanding of disease transmission. Sections 2 and 3 introduce the foundational SEIR and Erlang-distributed SEIR models, respectively. Readers familiar with these models may skip directly to Sect. 4. The subsequent sections focus on the proposed modifications, which explicitly model asymptomatic spread and its potential impact on disease outbreaks.

## The SEIR Model

**Fig. 1:**
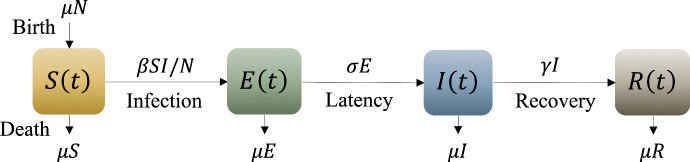
Four main compartments in the SEIR model with transition rates between compartments. Susceptible (*S*(*t*)): Individuals capable of contracting the disease. Exposed (*E*(*t*)): Individuals that are infected but not infectious. Infected (*I*(*t*)): Individuals capable of transmitting the disease. Recovered (*R*(*t*)): Those who are permanently immune (Color Figure Online)

In the SEIR model, individuals transition from the susceptible class (S) to the exposure class (E) upon contact with an infectious individual. The exposed individuals then progress to the infectious class (I) after a latent period, during which they are infected but not yet infectious. Finally, individuals in the infectious class (I) transition to the recovered class (R) after a duration of infectiousness. The transition in and out of the four classes, including a natural death/birth rate $\mu$, are shown in Fig. 1.

The model parameters and main model assumptions are,

- The population size is constant $N \equiv N(t) = S(t) +E(t) + I(t)+R(t)$, for any *t*.
- The disease is not lethal, and birth and death rates are assumed to equal $\mu$.
- The transmission parameter, $\beta$, is defined as the average number of *effective* contacts with other individuals per infectious per unit time. An *effective* contact is an encounter in which the infection is transmitted; we assume this has a probability *b*. Assuming the contacts per unit time is given by *k*, the transmission parameter is then given by $\beta = k b.$
- The recovery rate is $\gamma$, so that $1/\gamma$ is the mean infectious period.
- Similarly, the mean latent period is $1/\sigma$.

The governing equations for each of the categories are

1a $$\begin{aligned} \frac{dS(t)}{dt}= &  \mu \, N - \mu \, S(t) - kb \,\frac{I(t)\,S(t)}{N}, \end{aligned}$$

1b $$\begin{aligned} \frac{dE(t)}{dt}= &  - \mu \, E(t) + kb \,\frac{I(t)\,S(t)}{N} - m \,\sigma E(t),\end{aligned}$$

1c $$\begin{aligned} \frac{dI(t)}{dt}= &  - \mu \, I(t) + m\, \sigma E(t) - n\, \gamma \, I(t),\end{aligned}$$

1d $$\begin{aligned} \frac{dR(t)}{dt}= &  -\mu \, R(t) + n\, \gamma \,I(t). \end{aligned}$$

One of the fundamental assumptions SEIR models make is that the probability of transitioning between compartments is independent of the time spent in each compartment. This suggests that an individual who has recently been exposed has an equal likelihood of transitioning to the infectious category as an individual who has been exposed for a longer period. Not only is this incorrect in the general case, but it also relies heavily on the specific details of the disease being studied. One way to remove this assumption is by exploring an alternative probability distribution functions (PDFs) for the latent and infectious periods (Wearing et al. 2005; Chebotaeva and Vasquez 2023).

## Adding Erlang Distributions—The $$\hbox {SE}^m$$$$\hbox {I}^n$$R Model

From a mathematical perspective, assuming a constant transition rate between compartments implies that infectivity follows an exponential PDF. An exponential distribution represents the time between events in a Poisson process. Its core assumption is that events occur continuously and independently at a constant rate. This means that, in the exponential distribution, the likelihood of an event happening in the future is not influenced by the time that has already passed. This memoryless property inherent to exponential distributions makes them less ideal for epidemiological models, as factors like the duration of exposure or the individual’s immune state can influence the likelihood of infection.

**Fig. 2:**
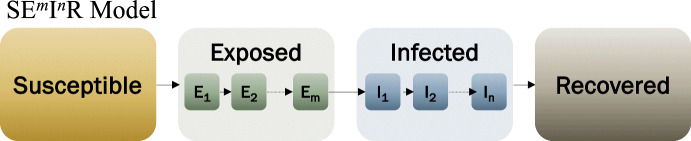
Erlang distributed SEIR model. Since we consider *m* sub-compartment for *E* and *n* sub-compartment for *I*, we denote this model as $\hbox {SE}^m$ $\hbox {I}^n$R(Color Figure Online)

There are various approaches to remove this condition. The two most common are (i) using integro-differential equations, (Kermack and McKendrick 1927; Hethcote and Tudor 1980; Keeling and Grenfell 1997; Feng et al. 2007; Cushing 2013), or (ii) subdividing the classes into sub-compartments, as in Lloyd (2001), Cunniffe et al. (2012), Sherborne et al. (2015), Chebotaeva and Vasquez (2023). The latest approach is also known as the “linear chain trick” approach (Cushing 1998; Hurtado and Kirosingh 2019; Lloyd 2001). In this study, we follow the multiple sub-compartment approaches, illustrated in Fig. 2, and based on the model proposed by Wearing et al. (2005); Chebotaeva and Vasquez (2023).

An Erlang distribution describes the probability distribution of the sum of *k* independent exponential random variables. Each exponential variable has a mean of $1/\lambda$, representing the average time between events. Alternatively, it can be viewed as the distribution of the time until the $k^{\text {th}}$ event occurs in a Poisson process with rate $\lambda$. The PDF for an Erlang distribution is then given by

2 $$\begin{aligned} f\left( x; k,\lambda \right) = \frac{\lambda ^k x^{k-1} e^{-\lambda x}}{(k-1)!}. \end{aligned}$$

As it can be deduced from Eq. (2). the Erlang distribution is a special case of the Gamma distribution where the shape parameter is the discrete variable, *k*.

An Erlang-distributed SEIR model has the time spent in the exposed and infectious classes modeled by an Erlang distribution with rates $\sigma$ and $\gamma$, and shape parameters *m* and *n*, respectively. As discussed in Fig. 2, we refer to this model as the $\hbox {SE}^m$ $\hbox {I}^n$R model.

**Fig. 3:**
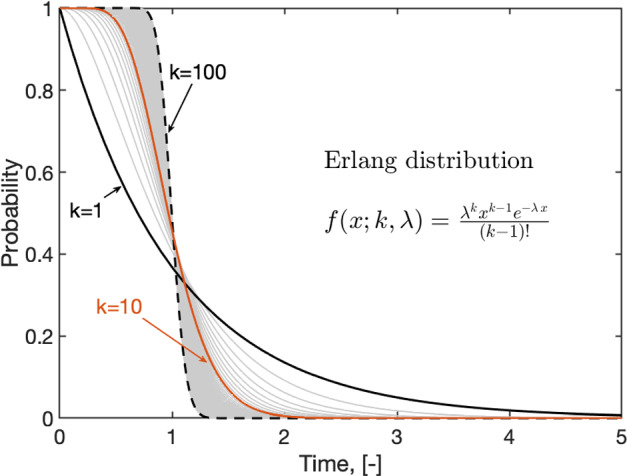
Probabilities given by an Erlang distribution with shape parameter *k* and rate $\lambda$. The PDF shown in the figure, $f(x; k,\lambda )$, is valid for $x,\lambda \ge 0$. In these examples, $\lambda = 1$. An Erlang distribution equals an exponential distribution when $k=1$ (Color Figure Online)

Figure 3 illustrates the expected probabilities of an Erlang-distributed variable as a function of the number of sub-compartments, $1 \le k < \infty$. At the lower bound, when the value of *k* is equal to 1, the probability density function simplifies to the exponential distribution. At the upper bounds, in the limit as *k* goes to infinity, the duration of stay in a class will be the same for all individuals. It is notable that the probabilities predominantly center on the rate, $\lambda = 1$ for this particular example. The differing factor is the extent of the surrounding spread; it is tighter for larger *k*’s. This implies that the main difference between exponential and Erlang-distributed disease dynamics is in the transients rather than in the averages and/or steady-state values. From an epidemiological perspective, the dynamics of disease transmission are highly significant due to their direct influence on resource allocation. We continue the discussion in Sect. 3.1, where we provide specific examples.

In addition to the assumptions made in the SEIR model, the $\hbox {SE}^m$ $\hbox {I}^n$R model has the following assumptions:

- The population size is constant and equal to $N = S+ \sum _{i=1}^{m} E^i+ \sum _{k=1}^n I^k +R$.
- The exposed class is divided into *m* sub-classes, and $m \sigma$ is the rate of sequential progression through the sub-classes, where $1/\sigma$ is the mean latent period. This is a proxy of modeling the latent period as a Gamma distribution with shape parameter *m* and rate parameter $\sigma$.
- Infected classes are divided into *n* sub-classes, and $n \gamma$ is the rate of sequential progression through the sub-classes, where $\gamma$ is the recovery rate so that $1/\gamma$ is the mean infectious period. As before, this corresponds to a Gamma distribution with shape parameter *n* and rate parameter $\gamma$.

The governing system of equations is given by,

3a $$\begin{aligned} \frac{dS(t)}{dt}= &  \mu \, N - \mu \, S(t) - kb\, \frac{I(t)\,S(t)}{N}, \end{aligned}$$

3b $$\begin{aligned} \frac{dE^1(t)}{dt}= &  - \mu \,E^1(t) + kb\, \frac{I(t)\,S(t)}{N} - m \,\sigma \, E^1(t),\end{aligned}$$

3c $$\begin{aligned} \frac{dE^{i}(t)}{dt}= &  - \mu \, E^i(t) + m\, \sigma \, \left[ E^{i-1}(t) - E^i(t)\right] , \hspace{1cm} i = 2,..., m,\end{aligned}$$

3d $$\begin{aligned} \frac{dI^1(t)}{dt}= &  - \mu \, I^1(t) + m\, \sigma \,E^m(t) - n \gamma \, I^1(t),\end{aligned}$$

3e $$\begin{aligned} \frac{dI^k(t)}{dt}= &  - \mu \, I^k(t) + n\, \gamma \,\left[ I^{k-1}(t) - I^{k}(t)\right] , \hspace{1cm} k = 2,..., n,\end{aligned}$$

3f $$\begin{aligned} \frac{dR(t)}{dt}= &  -\mu \, R(t) + n \,\gamma \, I^n. \end{aligned}$$

Many research studies have investigated the effects of using Erlang probability density functions (PDFs) on the transition rates in SEIR models (Krylova and Earn 2013; Wearing et al. 2005; Bolzoni et al. 2021; Carbone and De Vincenzo 2022; Meehan et al. 2023; Chebotaeva and Vasquez 2023). In general, they have found that this approach is sufficiently flexible to provide a good approximation of realistic distributions for latent and infectious periods (Krylova and Earn 2013; Wearing et al. 2005). It is worth mentioning that the differences between the SEIR and $\hbox {SE}^m$ $\hbox {I}^n$R models become even more noticeable when factoring in time-dependent effects on the transmission and evolution of the disease. For instance, we can consider factors like loss of immunity and seasonality, as discussed in the next section.

### $$\hbox {SE}^m$$$$\hbox {I}^n$$R Model with Loss of Immunity and Seasonality

**Fig. 4:**
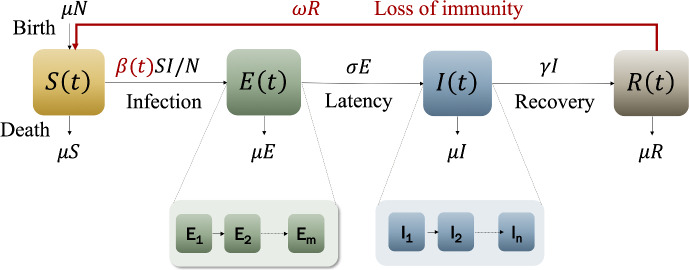
$\hbox {SE}^m$ $\hbox {I}^n$R model including loss of immunity at a rate $\omega$, and seasonality, captured by the time-dependent transmission parameter, $\beta (t)$ (Color Figure Online)

Figure 4 depicts the model resulting from adding loss of immunity to the $\hbox {SE}^m$ $\hbox {I}^n$R model. Here, we include a new parameter, $\omega$, which is the rate at which immunity is lost. In addition, to account for seasonality, we use a time-varying rate of infection, $\beta (t)$, given by,

4 $$\begin{aligned} \beta (t) =\frac{k\,b}{2}\,\left[ 1 + \beta _1 \cos \left( 2\pi \omega _S t\right) \right] , \end{aligned}$$

here, $0< \beta _1 < 1$ is the reduction percentage of $\beta$, and $\omega _S$ is the reciprocal of the average protected period specific to seasonality.

**Fig. 5:**
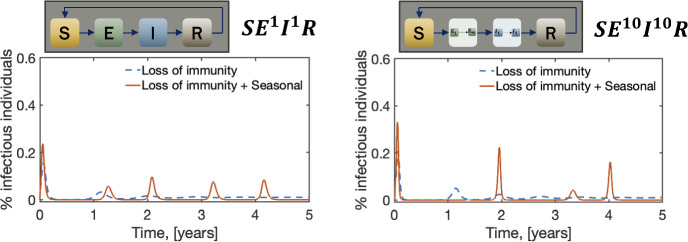
The differences between exponential and Erlang-distributed SEIR models become more noticeable when considering the loss of immunity and seasonality. Here *m* denotes the number of sub-compartments in class *E* and *n* the number of sub-compartments in class *I*. The standard SEIR model corresponds to $m=n=1$, and here we are comparing it with a $\hbox {SE}^m$ $\hbox {I}^n$R model with $m=n=10$. In addition, $\mu =(76\cdot 365)^{-1}$, $\omega _S =(365)^{-1}$ and $\beta _1= 0.75$. Other parameter values are given in Table 1 (Color Figure Online)

Figure 5, shows how the SEIR and $\hbox {SE}^m$ $\hbox {I}^n$R models differ when loss of immunity and seasonality are considered. The parameter values for these simulations are given in Table 1. With the basic SEIR model, we see a second peak in the infectious after one year due to the loss of immunity (blue dashed lines). This is mainly because in these simulations, $1/\omega = 365$ days. Once we include ten sub-compartments ($\hbox {SE}^{10}$ $\hbox {I}^{10}$R), the magnitude of that second peak increases from $\sim 3\%$ to $\sim 5\%$. This means that when we include loss of immunity, the SEIR model underestimates the number of infectious individuals after the initial peak.

When a seasonal component is incorporated into the basic SEIR model (represented by red solid lines), the resulting model exhibits periodic peaks in disease prevalence. These peaks have a consistent frequency and amplitude, creating a predictable pattern for the SEIR model. However, the introduction of seasonality in the $\hbox {SE}^m$ $\hbox {I}^n$R model disrupts this uniformity. Unlike the SEIR model, the $\hbox {SE}^m$ $\hbox {I}^n$R model with seasonality displays peaks that vary in both magnitude and timing. Additionally, similar to the impact of loss of immunity, seasonal forcing in the $\hbox {SE}^m$ $\hbox {I}^n$R model can lead to an overall increase in peak severity.

Perhaps the most significant consequence of this disruption is the potential for misleading predictions. For example, imagine allocating resources for disease control based on the predictable peak timing forecast by the SEIR model in year two. However, if the actual disease dynamics follow the more realistic yet irregular pattern predicted by the $\hbox {SE}^m$ $\hbox {I}^n$R model, such resource allocation could be ineffective and potentially lead to devastating consequences.

**Fig. 6:**
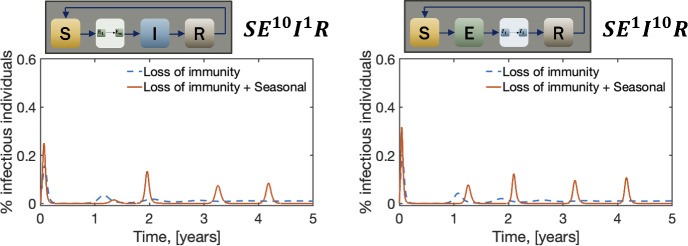
The differences between exponential and Erlang distributed SEIR models when the number of sub-compartments in the exposed and infectious classes varies separately. In this figure, $\mu =(76\cdot 365)^{-1}$, $\omega _S =(365)^{-1}$ and $\beta _1= 0.75$.Parameter values are given in Table 1 (Color Figure Online)

To investigate if these differences arise from adding sub-compartments to the exposed class, *m*, or the infectious class, *n*, we vary the number of sub-compartments in these two classes separately. In Fig. 6, we show these results. The figure indicates that the peak around one year is lost due to the inclusion of sub-compartments in the exposed class, while the increase in the magnitude comes from adding sub-compartments to either the exposed or the infectious classes. These findings suggest that accurately capturing the distribution of infectiousness and the latent period, achieved through Erlang distributions in the $\hbox {SE}^m$ $\hbox {I}^n$R model, plays a crucial role in not only the magnitude of the outbreaks but also their timing. This highlights the importance of considering both Erlang-distributed infectious and latent periods when modeling diseases with seasonal forcing.

The results presented in Figs. 5, 6 consistently demonstrate that relying on constant transition rates instead of Erlang distributions underestimates the dynamic nature of disease progression. These discrepancies become even more pronounced when we factor in loss of immunity and seasonal variations. Interestingly, the equilibrium points remain relatively unaffected.

The stark differences observed between the SEIR and $\hbox {SE}^m$ $\hbox {I}^n$R models highlight the need to refine the underlying assumptions. In the following sections, we address this by incorporating a broader range of social and behavioral factors that influence the spread of epidemics.

## New Modeling Considerations

This manuscript explores the effects of differentiating exposed individuals based on whether they had contact with symptomatic or asymptomatic infectious individuals. Understanding this distinction is crucial for developing effective disease control strategies. By analyzing transmission patterns and outcomes among those exposed to symptomatic or asymptomatic individuals, we can gain valuable insights into disease dynamics and the potential role of asymptomatic spread. This research aims to provide public health officials, policymakers, and healthcare professionals with valuable information to implement targeted interventions and preventive measures, mitigating the risk of transmission from both symptomatic and asymptomatic individuals.

In prior studies, the infectious class (*I*) has been divided into symptomatic ($I_s$) and asymptomatic ($I_a$) sub-classes (Wearing et al. 2005; Peirlinck et al. 2020). However, in those studies, the exposed (*E*) class did not distinguish between exposure to symptomatic or asymptomatic individuals. The latest data from COVID-19 datasets reveal that the chances of developing symptoms and becoming infectious vary for individuals who interact with symptomatic or asymptomatic individuals (Methi and Madslien 2022). To resolve this, we suggest subdividing the *E* class into two distinct categories: Exposed from an asymptomatic source ($E_a$) and exposed from contact with a symptomatic individual ($E_s$). In addition, we include delayed dynamics to account for self-quarantine and isolation, where susceptible individuals who have been in contact with infectious individuals symptomatic enter a susceptible quarantined class ($S_q$) after a delay ($t_q$). This delay reflects real-world constraints, such as the time taken for contact tracing and testing, which involves identifying individuals who have been in close contact with a known infectious person. They are then quarantined to prevent further spread of the disease, even before they show symptoms or are confirmed as infected. Once quarantined, individuals remain isolated for a fixed period before returning to the susceptible pool. This approach assumes ideal quarantine, aligning with prior work that highlights how ideal quarantine provides theoretical upper bounds for intervention efficacy (Browne et al. 2015). “Ideal” implies that there is full isolation and compliance with the quarantine.

We also note that different studies have included quarantine and isolation in their models. In particular, the study by Heideicke et al. (2024) examined scenarios where infections are confirmed through testing undetected infectious individuals. These individuals are assumed to self-isolate and identify infected contacts, who can then be traced and quarantined either during their latent period or after becoming infectious. The study also divided the infectious stage into early and late phases but did not distinguish between symptomatic and asymptomatic cases.

**Fig. 7:**
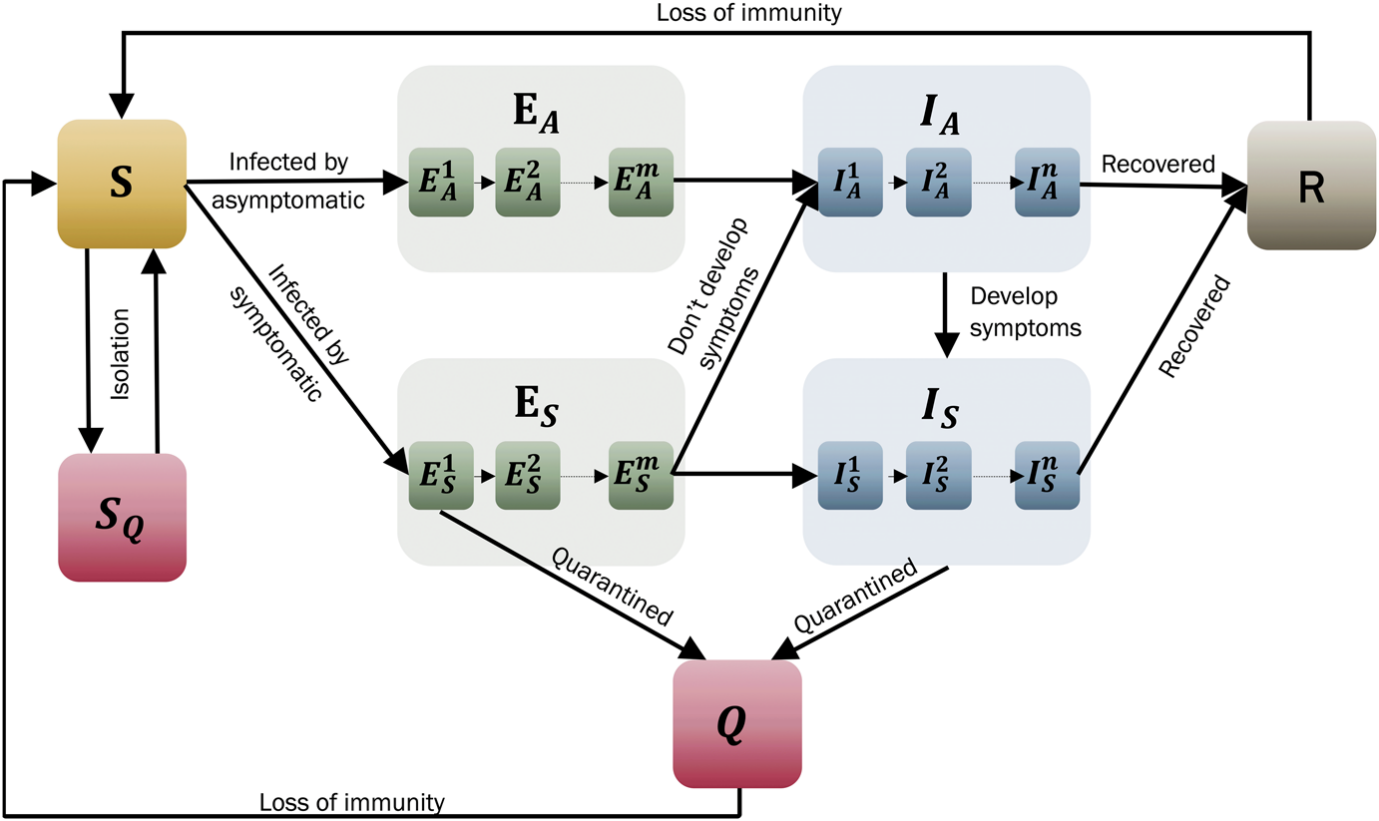
Proposed $\hbox {SE}_{\text {a,s}}^m$ $\hbox {I}_{\text {a,s}}^n$R model, including self-isolation, quarantine and loss of immunity (Color Figure Online)

As a first step, our model assumes ideal self-quarantine and isolation. While acknowledging that real-world quarantine measures are imperfect and could be addressed by incorporating a quarantine infection rate or partial compliance (Browne et al. 2015), we defer these considerations to future work. The resulting model is illustrated in Fig. 7.

Following the same basic assumptions from the $\hbox {SE}^m$ $\hbox {I}^n$R model and the transition dynamics shown in Fig. 7, the resulting system of equations is,

$$\begin{aligned} \frac{dS}{dt}= &  - \frac{k b_{_A}}{N} I_A(t) S(t)- \frac{k b_{_S}}{N} I_S(t) S(t) - \frac{qk\left[ 1- b_{_S} \right] }{N} I_S(t) S(t) \\  &  + \frac{qk\left[ 1- b_{_S} \right] }{N} I_S(t-\tau _{_Q}) S(t-\tau _{_Q}) + \omega R(t) + \omega Q(t)\\ &  \\ \frac{dS_Q}{dt}= &  \frac{qk\left[ 1- b_{_S} \right] }{N} I_S(t) S(t) - \frac{qk\left[ 1- b_{_S} \right] }{N} I_S(t-\tau _{_Q}) S(t-\tau _{_Q}) \\ &  \\ \frac{dE^1_A}{dt}= &  \frac{k b_{_A}}{N} I_A(t) S(t) - m \sigma E^1_A(t)\\ &  \\ \frac{dE^i_A}{dt}= &  m \sigma E^{i-1}_A(t) - m \sigma E^i_A(t), \hspace{2cm} i = 2, \dots , m\\ &  \\ \frac{dE^1_S}{dt}= &  \frac{k b_{_S}}{N} I_S(t) S(t) - \frac{k b_{_S} q}{N} I_S(t) S(t) - m \sigma E^1_S(t)\\ &  \\ \frac{dE^i_S}{dt}= &  m \sigma E^{i-1}_S(t) - m \sigma E^i_S(t), \hspace{2cm} i = 2, \dots , m-1\\ &  \\ \frac{dE^m_S}{dt}= &  m \sigma E^{m-1}_S(t) - \left[ 1- \kappa \right] m \sigma E^m_S(t) - \kappa m \sigma E^m_S(t)\\ &  \\ \frac{dI_A^1}{dt}= &  m \sigma E^m_A(t) + \kappa m \sigma E^m_S(t) - n \gamma I^1_A(t) - P_I^1(t)\\ &  \\ \frac{dI_A^i}{dt}= &  n \gamma I^{i-1}_A(t)- n \gamma I^i_A(t) - P_I^i(t), \hspace{2cm} i = 2, \dots , n\\ &  \\ \frac{dI_S^1}{dt}= &  P_I^1(t) + \left[ 1- \kappa \right] m \sigma E^m_S(t) - n \gamma I_S^1(t) - d_I I_S^1(t)\\ &  \\ \frac{dI^i_S}{dt}= &  P_I^i(t) + n \gamma I_S^{i-1}(t) - n \gamma I_S^i(t) - d_I I_S^i(t), \hspace{2cm} i = 2, \dots , n\\ &  \\ \frac{dQ}{dt}= &  \frac{k b_{_S} q}{N} I_S(t) S(t) + d_I I_S(t) - \omega Q(t) \\ &  \\ \frac{dR}{dt}= &  n \gamma I_A^n(t) + n \gamma I_S^n(t) - \omega R(t) \end{aligned}$$

where

$$\begin{aligned} P_I^i(t) = m \sigma \kappa \, E_A^m\left( t -\tau _{_D}\right) \frac{\left[ n \gamma \tau _{_D}\right] ^{i-1}}{\left[ i-1\right] !}\, \exp \left[ -n \gamma \tau _{_D}\right] . \end{aligned}$$

The parameter $\tau _q$ represents the average time delay between contact tracing and the initiation of quarantine. This could result from inefficiencies in tracking or tracing individuals. The parameter *q* indicates the proportion of susceptible individuals identified for quarantine after contact with symptomatic cases. While *q* is set to 0.5 in our base model, studies suggest that increasing *q* through enhanced contact tracing could significantly reduce transmission, especially in high-incidence settings (Browne et al. 2015). In the $\hbox {I}_A$ class, we consider both asymptomatic and pre-symptomatic cases of infection. Both types of individuals can transmit the disease without displaying symptoms. However, the crucial difference lies in the development of symptoms. Asymptomatic cases are characterized by the absence of symptoms throughout the entire duration of the infection. On the other hand, individuals who are pre-symptomatic are in the initial phase of infection and have exhibited no symptoms. Eventually, these individuals will exhibit symptoms, but they can still transmit the virus before symptoms appear.

In the model, the proportion of pre-symptomatic cases is controlled by $P_I^i(t)$. This variable represents the rate of transition of individuals from the asymptomatic infectious class $\hbox {I}^i_A$ to the symptomatic infectious class $\hbox {I}^i_S$. In other words, $P_I^i(t)$ calculates the inflow of individuals into the $i^{\text {th}}$ infectious symptomatic class at time *t* who were previously exposed to asymptomatic cases at different stages ($\hbox {E}^1_A$ to $\hbox {E}^m_A$) some time ago $\left( t - \tau _{_D}\right)$, weighted by their infectiousness and the probability of remaining undiagnosed until the current time. The binomial coefficient was introduced to account for the fact that higher stages likely contribute more due to a potentially higher viral load closer to becoming infectious. This modeling approach is particularly applicable to pathogens with aerial transmission, where the quantity of viral particles released into the environment by an infected individual is directly proportional to their viral load (Gandolfi et al. 2015).

While our model considers the impact of viral load on disease progression and transmission, it does not explicitly model the direct relationship between the quantity of viral load and transmission. To explore this correlation, alternative modeling approaches like immuno-epidemiological infection-age or integro-differential equation models could provide valuable insights for various transmission modes, including airborne and other routes of infection, as discussed in Gandolfi et al. (2015).

Finally, we note that, for simplicity, we have neglected the natural birth/death rate, $\mu = 0$. Other parameter descriptions and values are given in Table 1. In addition, we performed Partial Rank Correlation Coefficients (PRCC) analysis to quantify the influence of individual model parameters on the model’s outputs. The results of this sensitivity analysis are presented in Appendix A.

**Table 1:** Model Parameters. All time units are given in days

Param.	Description	Value	Conds.	Refs.
$k$	Number of contacts per unit time	$1.41$	$k>0$	Jafar Hasanzadeh and Mirahmadizadeh (2022)
$b_{_S}$	Probability of transmitting the disease for a symptomatic individual	$0.316$	$0\le b_{_S} \le 1$	Agrawala and Bhardwaj (2021)
$b_{_A}$	Probability of transmitting the disease for an asymptomatic individual	$0.0316$	$0\le b_{_A} \le 1$	
$m$	Number of classes in the exposed class	20	$m \in {\mathbb {N}}$	
$1/\sigma$	Mean latent time	5.5	$\sigma >0$	Xin et al. (2022)
$n$	Number of classes of infectious class	3	$n \in {\mathbb {N}}$	
$1/\gamma$	Mean recovery time	6	$\gamma >0$	Rakshit et al. (2022)
$\kappa$	Percent of people who become asymptomatic after being infected by a symptomatic individual	0.15	$0\le \kappa \le 1$	Methi and Madslien (2022)
$\omega$	Rate of loss of immunity	$(365)^{-1}$	$0\le \omega \le 1$	
$q$	Percent of people who had contact with infectious and symptomatic individuals but did not contract infection who were quarantined in $Sq$ (quarantined susceptible class)	0.5	$0\le q \le 1$	
$\tau _{_Q}$	Days spent in quarantine	10	$\tau _{_Q} \ge 0$	https://archive.cdc.gov/www_cdc_gov/coronavirus/2019-ncov/hcp/duration-isolation.html#:~:text=Isolation%20can%20be%20discontinued%20at,and%20other%20symptoms%0are%20improving
$d_i$	Daily rate of isolation of newly infectious cases	0.2	$0\le d_i \le 1$	
$\tau _{_D}$	Delay, which accounts for the incubation period for individuals who were exposed to asymptomatic cases	7	$0 \le \tau _{_D} \le t_q$	
$\mu$	Birth/death rate	0	$0 \le \mu \le 1$	$\left( ^*\right)$
$N$	Total Population	$1000$	$N \in {\mathbb {N}}$	
$I_0$	Initial number of infected	N/10	$I_0 \in {\mathbb {N}}$	
$S_0$	Initial number of susceptible	$N - I_0$	$S_0 \in {\mathbb {N}}$	

$\left( ^*\right)$ A different value for this parameter was used in Sect. 3.1. The value is given in the caption of Figs. 5, 6

## Results

### The Impact of Asymptomatic Cases on Self-isolation Strategies

**Fig. 8:**
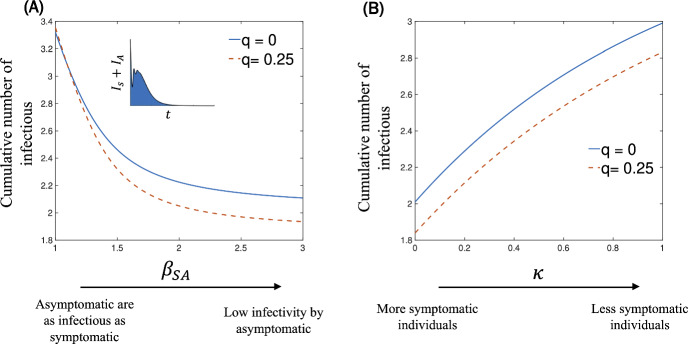
Cumulative number of infectious individuals as a function of (**A**) the relative infectivity of asymptomatic individuals, $\beta _{{SA}}$ and (**B**) percentage of individuals exposed to symptomatic infectious individuals who do not develop symptoms, $\kappa$. Comparison between no self-isolation of individuals who came into contact with symptomatic individuals (solid - blue lines) and the case in which 25% of those individuals self-isolate (dashed - red lines). The cumulative number is calculated as the area under of the curve of $I_s + I_A$ versus time (Color Figure Online)

In Fig. 8, we investigate the efficacy of self-isolation strategies of susceptible individuals following contact with symptomatic, infectious individuals. To do this, we vary the two main parameters that distinguish symptomatic and asymptomatic infections in our model, $\beta _{{SA}}$ and $\kappa$.

The first parameter, $1\le \beta _{{SA}} \le { 3}$, represents the relative infectiousness of asymptomatic individuals compared to their symptomatic counterparts. Mathematically, if $b_{_S}$ denotes the probability of transmission from a symptomatic individual, the corresponding probability for asymptomatic individuals is $b_{_A} = b_{_S}/\beta _{{SA}}$. In essence, $\beta _{{SA}}$ quantifies the degree of attenuation in infectiousness associated with asymptomatic cases. The second parameter, $0 \le \kappa \le 1$, captures the proportion of exposed individuals who do not develop symptoms, even after they are exposed to symptomatic infectious individuals. This metric effectively captures the prevalence of asymptomatic cases arising from exposure to symptomatic individuals.

Figure 8A shows that implementing self-isolation strategies effectively reduces the total number of infectious individuals. However, when the transmission rate of asymptomatic individuals approaches that of symptomatic individuals ($\beta _{{SA}}\sim 1$), self-isolation measures become ineffective, resulting in the same number of infectious individuals as in the absence of any intervention. As $\beta _{{SA}}$ increases, the impact of self-isolation becomes more pronounced. This observation aligns with the model’s assumptions: When $\beta _{{SA}}\simeq 1$, the infectiousness of both symptomatic and asymptomatic individuals becomes equivalent. Consequently, isolation interventions triggered solely by contact with symptomatic individuals have a lower impact on the overall infectious period. This finding highlights the critical role of asymptomatic infectiousness in shaping the effectiveness of self-isolation strategies.

Similarly, Fig. 8B shows that the number of infectious individuals increase with increasing values of $\kappa$. This is because a higher $\kappa$ value indicates a greater number of individuals who are infected but exhibit no symptoms. These asymptomatic individuals can unknowingly transmit the disease to others, contributing to a higher overall infection count. However, the effectiveness of isolation strategies remains relatively constant, irrespective of the value of $\kappa$. This is because isolation primarily targets symptomatic individuals.

In Summary, Fig. 8 emphasizes the importance of addressing asymptomatic individuals in infectious disease control. While self-isolation is effective against symptomatic cases, its impact is reduced when asymptomatic individuals are highly infectious or prevalent. This means that to optimize control strategies, it’s crucial to consider the specific characteristics of the pathogen and the population.

### Interplay Between Mean Recovery Time and Asymptomatic Spread

**Fig. 9:**
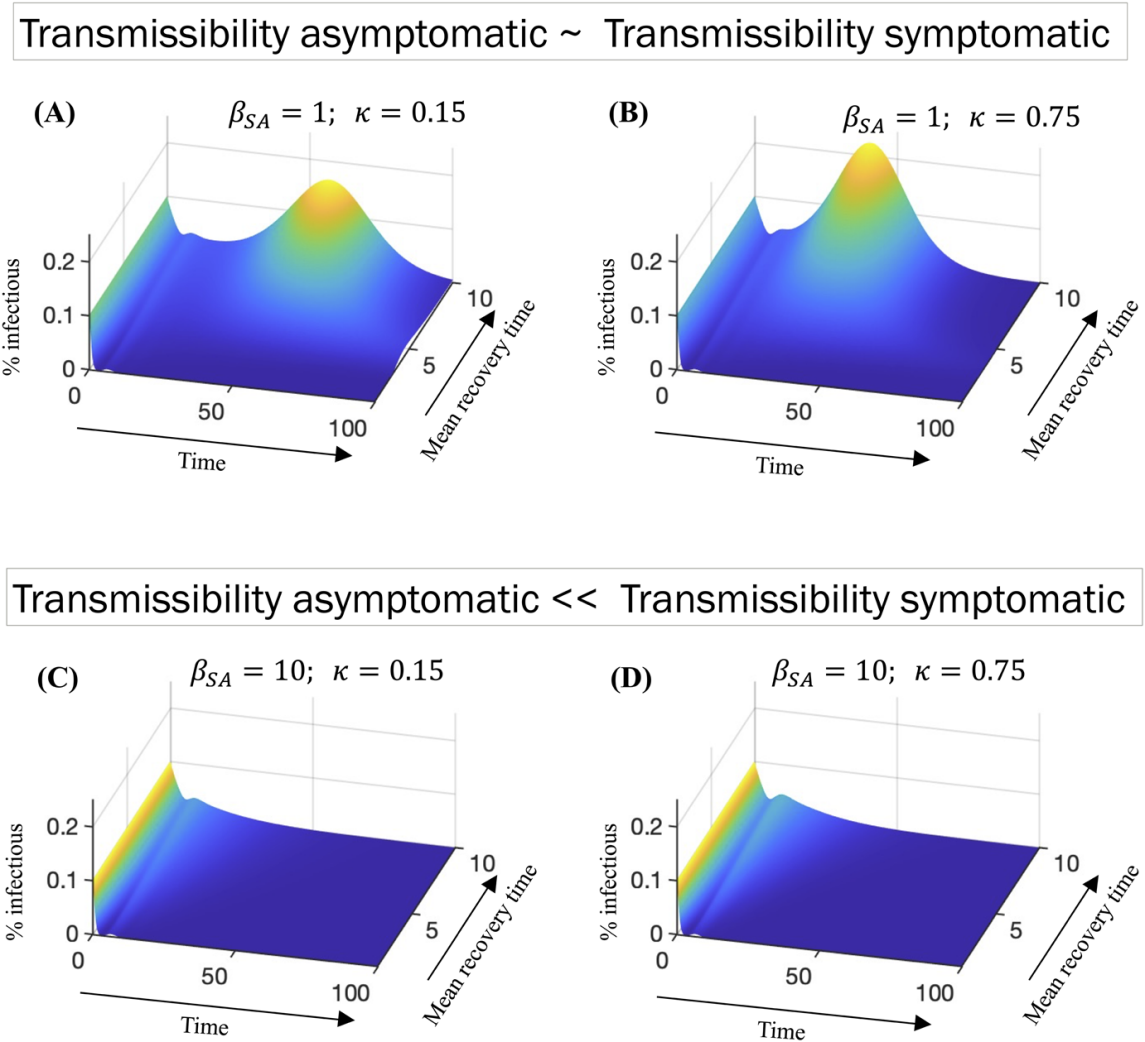
Percentage of infectious individuals (I = $\hbox {I}_A$ + $\hbox {I}_S$) as a function of time and mean recovery time, $1/\gamma$. In this figure, we assume that 50% of susceptible individuals (S) self-isolate after contact with symptomatic, infectious individuals ($\hbox {I}_S$). Plots (**A**) and (**B**) correspond to the case in which asymptomatic are as infectious as symptomatic individuals, $\beta _{{SA}}=1$. Plots (**C**) and (**D**) correspond to the case in which symptomatic individuals are ten times more infectious than asymptomatic individuals, $\beta _{{SA}}={10}$. The parameter $\kappa$ represents the percentage of individuals who are exposed to symptomatic individuals who become infectious but do not develop symptoms (Color Figure Online)

Figure 9 illustrates how the time evolution of the percentage of infectious individuals varies with the mean recovery time for different values of the relative infectiousness, $\beta _{{SA}}$, and the proportion of individuals who become asymptomatic, $\kappa$. A key insight gained from comparing the upper and lower panels of the figure is that the level of transmissibility associated with asymptomatic individuals can significantly alter the course of the epidemic.

In Fig. 9, plots (A) and (B) reveal a late peak in infections when the infectivity of asymptomatic individuals is comparable to symptomatic cases, $\beta _{{SA}}=1$. The magnitude of this peak increases with longer mean recovery times and higher values of $\kappa$. Moreover, an increase in $\kappa$ leads to an earlier occurrence of this peak. On the other hand, plots (C) and (D) examine the scenario where the infectivity of asymptomatic individuals is significantly lower than that of symptomatic individuals $\beta _{{SA}}=10$. In this case, the late peak is eliminated due to the presence of quarantine and isolation measures, which effectively control the peak when the relative infectiousness of asymptomatic individuals is low.

In general, Fig. 9 underscores the critical interplay between mean recovery time and the timing of isolation and quarantine measures. Early identification and isolation of both symptomatic and asymptomatic cases are crucial for a quicker reduction in the peak prevalence of infections. However, the figure also highlights limitations, particularly when dealing with highly infectious asymptomatic cases. This emphasizes the need to explore additional public health interventions, especially in scenarios with longer mean recovery times and situations where asymptomatic individuals are highly infectious.

### Loss of Immunity and Asymptomatic Impact on Equilibria

**Fig. 10:**
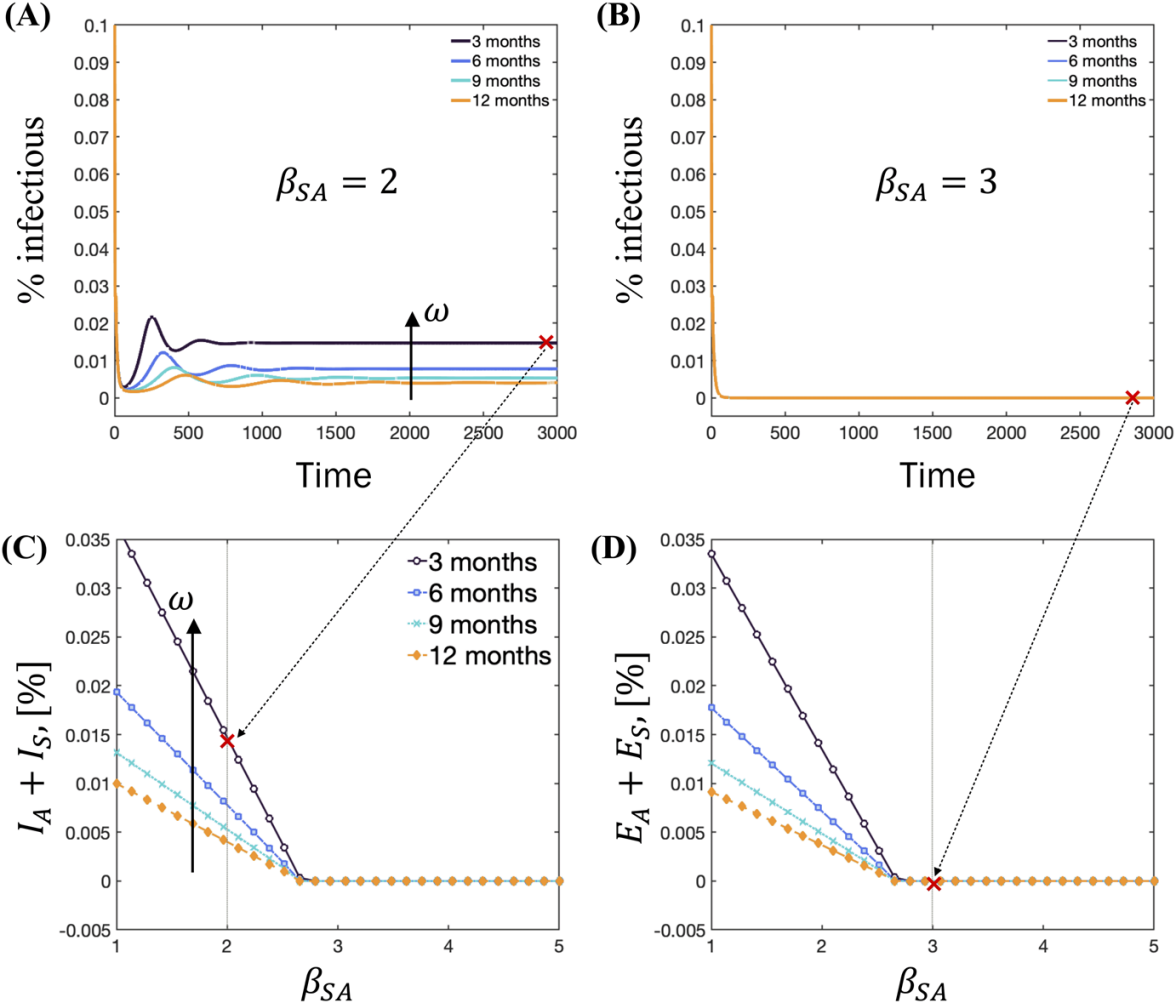
Time evolution of percentage of infectious individuals, I = $\hbox {I}_S$ + $\hbox {I}_A$, for (**A**) $\beta _{{SA}}=2$ and (**B**) $\beta _{{SA}}= 3$. From these figures we obtain the equilibrium points for each parameter combination. The equilibrium points, as a function of the relative infectiousness parameter, $\beta _{{SA}}$ and rate of loss of immunity, $\omega$, are given for (**C**) total infectious individuals, I = $\hbox {I}_A$ + $\hbox {I}_S$, and (**D**) total exposed individuals, E = $\hbox {E}_A$ + $\hbox {E}_S$ (Color Figure Online)

The previous sections assumed no loss of immunity, $\omega = 0$, leading to a disease-free equilibrium as the only stable state. However, introducing waning immunity, $\omega >0$, modifies these dynamics. As shown in Fig. 10A, endemic equilibria emerge at low values of the relative infectiousness parameter $\beta _{{SA}}$. In contrast, for larger values, the only equilibrium point is the disease-free equilibrium, where the percentage of infectious individuals approaches zero, as depicted in Fig. 10B.

In Fig. 10C, D, the equilibrium points are plotted as functions of the parameters $\beta _{{SA}}$ and $\omega$. These plots reveal a linear relationship between the equilibrium points and $\beta _{{SA}}$ when $\beta _{{SA}} \lesssim 2.7$. However, for $\beta _{{SA}} \gtrsim 2.7$, the system converges to the disease-free steady state. This critical threshold underscores the system’s sensitivity to variations in $\beta _{{SA}}$.

The underlying reason for this behavior is that $\beta _{{SA}} \approx 1$ indicates highly infectious asymptomatic individuals. In such cases, isolation and quarantine strategies, which primarily target symptomatic individuals, are less effective in curbing the spread of the disease. Conversely, a disease-free equilibrium can be achieved for higher $\beta _{{SA}}$ values due to the limited impact of asymptomatic transmission, coupled with the efficacy of isolation and quarantine measures in controlling symptomatic transmission.

In addition, higher values of the loss of immunity parameter, $\omega$, mean that individuals lose their immunity faster. This results in a larger group of susceptible individuals, leading to a larger number of exposed individuals, as shown in Fig. 10D. These dynamics allow the disease to persist at a higher level within the endemic equilibrium, challenging efforts to control the spread.

In summary, Fig. 10 illustrates the impact of waning immunity on disease dynamics. When highly infectious asymptomatic individuals are prevalent, the figure highlights the emergence of non-endemic equilibria, emphasizing the need for interventions targeting both symptomatic and asymptomatic populations. Conversely, an increased rate of immunity loss leads to a larger susceptible population, resulting in elevated numbers of infected and infectious individuals. This scenario necessitates the exploration of alternative control strategies.

### Relative Infectiousness Parameter Impact on Equilibria According to Different Disease Parameters and Control Measures

Infectious and latent periods are inherent characteristics of a disease that are beyond our control. However, the parameters $\beta$ and *q* can be managed to influence disease dynamics. This section explores how variations in these controllable parameters, along with changes in the relative infectiousness parameter $\beta _{{SA}}$, affect the equilibrium states of the system.

**Fig. 11:**
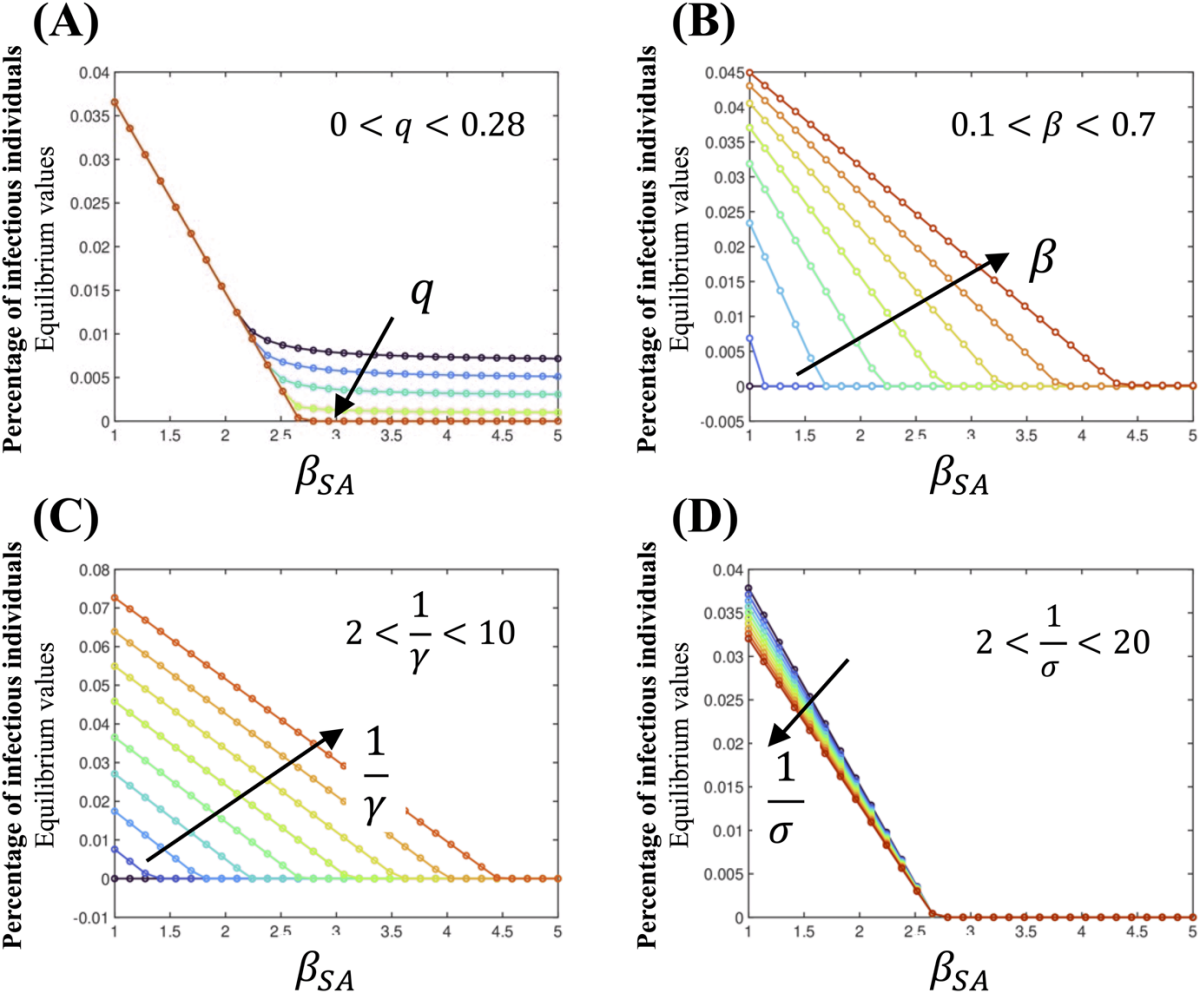
Equilibrium points as a function of the relative infectiousness parameter, $\beta _{{SA}}$ for (**A**) quarantine percentage *q*, (**B**) rate of infection $\beta$, (**C**) mean recovery time $1/\gamma$, and (**D**) mean latent time $1/\sigma$ (Color Figure Online)

While not shown in the figure, when $\kappa = 0$, the only equilibrium point corresponds to the disease-free state. For $\kappa >0$, the equilibrium points are independent of $\kappa$ and, in general, show a linear dependence on $\beta _{{SA}}$ for small values of the parameter, as shown in Fig. 11.

Figure 11A investigates the impact of the percentage of susceptible individuals who self-quarantine after exposure to a symptomatic infectious individual, as regulated by the parameter *q*. The figure shows that a disease-free equilibrium is unattainable for $q \lesssim 0.28$. For values of $q \gtrsim 0.28$, we found that all equilibrium points are independent of *q* and follow the same behavior captured by the empirical relation,5Figure 11B explores the effect of the transmission parameter $\beta$. The figure shows that increasing $\beta$ leads to a higher proportion of infectious individuals at equilibrium. However, when $\beta _{{SA}}$ is sufficiently high, the impact of $\beta$ on the equilibrium states becomes negligible, and the system converges to the disease-free equilibrium.

Figure 11C and D investigate the impacts of mean recovery and latent periods, respectively. A prolonged recovery period correlates with a higher percentage of infectious individuals at equilibrium. In contrast, variations in latent period have a negligible effect on the equilibrium proportion of infectious individuals. Intriguingly, an increased latent period leads to a lower equilibrium level of infection.

In summary, Fig. 11 demonstrates the influence of various factors on disease dynamics. While inherent biological characteristics like latent and recovery periods cannot be modified, parameters such as the transmission rate, $\beta$, and self-quarantine rate, *q*, can be strategically adjusted to impact disease spread. The figure highlights the critical role of self-quarantine in preventing disease outbreaks, as evidenced by the threshold value of *q*. Moreover, the relationship between the relative infectiousness of asymptomatic individuals, $\beta _{{SA}}$, and the overall transmission rate, $\beta$, is complex, with potential implications for disease control strategies. To effectively mitigate disease spread, it is essential to consider individual behaviors, including adherence to preventive measures and personal responsibility, as these factors significantly influence infection rates.

## Discussion and Conclusions

A new Erlang-distributed SEIR model has been developed, which considers the spread of an infectious disease through symptomatic and asymptomatic individuals. This new model offers valuable insights for public health strategies, as it provides a more nuanced understanding of disease transmission.

The main factor determining the difference between asymptomatic and symptomatic cases is the parameter $\kappa$. In the model, individuals are more likely to develop symptoms if the value of $\kappa$ is smaller. The model shows that lower values of $\kappa$ correspond to a slower spread of the disease, with a delayed and reduced peak of infections.

The findings underscore that the efficacy of isolation measures is highly dependent on the relative infectiousness of asymptomatic individuals $\beta _{{SA}}$ and the proportion of exposed individuals who become asymptomatic $\kappa$. Lower values of $\beta _{{SA}}$ and $\kappa$ lead to a notable reduction in both the peak magnitude and duration of the infectious period, highlighting the importance of these parameters in the prediction of the spread of the disease and thinking through isolation measures.

The interplay between mean recovery time and isolation measures plays a critical role in the temporal dynamics of the infection spread. Early and comprehensive isolation of symptomatic and asymptomatic individuals can significantly mitigate the peak prevalence of diseases. However, highly infectious asymptomatic cases represent a serious problem requiring additional public health measures to achieve effective control.

The study also delves into the implications of waning immunity, revealing that the emergence of endemic equilibria is contingent on the relative infectiousness of asymptomatic individuals. A higher rate of immunity loss $\omega$ leads to a larger susceptible population. These insights highlight the need for effective strategies that address both symptomatic and asymptomatic transmissions and consider the dynamics of immunity loss.

Overall, the model emphasizes the significance of understanding the distinct roles that symptomatic and asymptomatic individuals play in disease spread. It suggests that public health strategies should consider these differences to optimize the timing and scale of their responses, ultimately improving the management of healthcare resources and reducing the socio-economic impact of the disease.

Future considerations for this model could include incorporating additional factors influencing disease spread. For instance, the model could be expanded to account for spatial dynamics, where the location of individuals and their interactions play a role in transmission, similar to the study we presented in Chebotaeva and Vasquez (2023). Additionally, factors like age distribution, pre-existing immunity levels in the population, and the potential for mutations in the disease could be integrated to create a more comprehensive picture. Furthermore, the model’s predictions could be validated through real-world data analysis to assess its accuracy and refine its parameters.

## Figures and Tables

**Fig. 12:**
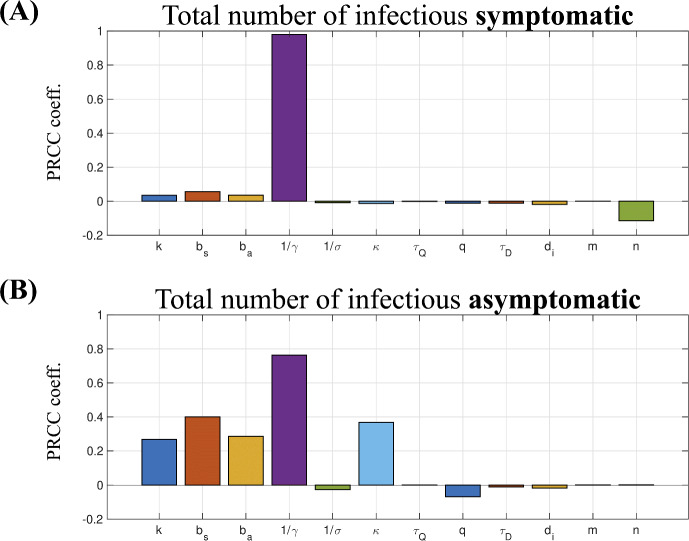
PRCC test for model parameters. Parameter descriptions and ranges are given in Table 2 (Color Figure Online)

## Appendix A Sensitivity Analysis

To assess the sensitivity of the model to parameter variations, we performed a partial rank correlation coefficient (PRCC) analysis. This analysis involved using Latin Hypercube Sampling (LHS) to generate 20,000 combinations of parameters within the ranges specified in Table 2. Figure 12 illustrates the sensitivity of the total cumulative number of infected individuals (both symptomatic and asymptomatic) after 200 time steps to each parameter.

The PRCC analysis reveals that the mean recovery time ($1/\gamma$) has the largest impact on the number of infected individuals. This is not unexpected since longer infectious periods increases the chances of transmission. The second most impactful parameter is the number of sub-compartments in the infectious classes (*n*). This underscores the importance of using an Erlang distribution, rather than an exponential distribution, when modeling infectious diseases.

In asymptomatic cases, the total cumulative infections are positively affected by several parameters besides the average recovery time ($1/\gamma$). These include the contact rate (*k*), the relative transmissibility of asymptomatic individuals ($\beta _{{SA}} = b_s/b_a$), and the proportion of individuals who become asymptomatic ($\kappa$). Finally, we note that, in contrast to its effects on infectious symptomatic individuals ($I_S$), the quarantine parameter (*q*) has a substantially more significant effect on the transmission dynamics within the infectious asymptomatic population ($I_A$).

**Table 2:** Parameter ranges for PRCC test

Param.	Description	Range
$k$	Number of contacts per unit time	[0.1, 0.5]
$b_{_S}$	Probability of transmitting the disease for a symptomatic individual	[0.7, 2]
$\beta _{{SA}}$	Relative infectiousness of asymptomatic individuals compared to symptomatic individuals, $\beta _{{SA}} = b_S/b_A$	[1, 5]
$1/\gamma$	Mean recovery time	[2, 10]
$1/\sigma$	Mean latent time	[2, 10]
$\kappa$	Percent of people who become asymptomatic after being infected by a symptomatic individual	[0.1, 0.5]
$\tau _{_Q}$	Days spent in quarantine	[2, 10]
$q$	Percent of people who had contact with infectious and symptomatic individuals but did not contract infection who were quarantined in $Sq$ (quarantined susceptible class)	[0.1, 0.3]
$\tau _{_D}$	Delay, which accounts for the incubation period for individuals who were exposed to asymptomatic cases	[2, 10]
$d_i$	Daily rate of isolation of newly infectious cases	[0.1, 0.3]
$m$	Number of classes in the exposed class	[3, 20]
$n$	Number of classes of infectious class	[3, 20]
